# Lysozyme gene treatment in testosterone induced benign prostate hyperplasia rat model and comparasion of its’ effectiveness with botulinum toxin injection

**DOI:** 10.1590/S1677-5538.IBJU.2016.0677

**Published:** 2017

**Authors:** Osman Ergün, Pinar Aslan Koşar, İbrahim Onaran, Hakan Darici, Alim Koşar

**Affiliations:** 1 Department of Urology, Konya Training and Research Hospital, Konya, Turkey; 2 Department of Medical Biology and Genetic, Süleyman Demirel University, Isparta, Turkey; 3 Deparment of Histology and Embryology, Süleyman Demirel University, Isparta, Turkey; 4 Department of Urology, Süleyman Demirel University, Isparta, Turkey

**Keywords:** Prostatic Hyperplasia, Botulinum Toxins, Testosterone

## Abstract

**Objectives:**

To compare the effects and histopathological changes of botulinum neurotoxin type A and lysozyme gene injections into prostate tissue within a testosterone induced benign prostate hyperplasia rat model.

**Materials and Methods:**

40 male Wistar rats were randomized into four Groups. Group-1: Control, Group-2: Testosterone replacement, Group-3: Testosterone+botulinum neurotoxin type A, Group-4: Testosterone+plazmid DNA/liposome complex.

**Results:**

Estimated prostate volume of the testosterone injected Groups were higher than the control (p <0.05). Actual prostate weight of the testosterone injected Groups was higher than the control Group (p <0.05). Testosterone undecanoate increased the prostate weight by 39%. Botulinum neurotoxin type A treatment led to an estimated prostate volume and actual prostate weights decreased up to 32.5% in rats leading to prostate apoptosis. Lysozyme gene treatment led to an estimated prostate volume and actual prostate weights decrease up to 38.7%.

**Conclusion:**

Lysozyme gene and botulinum neurotoxin type A treatments for prostate volume decreasing effect have been verified in the present study that could be anew modality of treatment in prostatic benign hyperplasia that needs to be verified in large randomized human experimental studies.

## INTRODUCTION

The most frequent cause of lower urinary tract symptoms (LUTS) is benign prostate hyperplasia (BPH). Increase in BPH incidence with age raises concern for both health and economy policies (1). The current medical treatments of BPH have some adverse effects such as dizziness, asthenia, postural hypotension in patients taking α-adrenergic antagonists, and decreased libido and impotence in patients taking 5α-reductase inhibitors. Therefore, researches are still in progress leading to constantly increasing costs of medical companies and health organizations.

Intra-prostatic botulinum neurotoxin type A (BoNT-A) injection is among novel treatment options of BPH in several rat, dog, and human studies (2-6). The prostate is a secretory organ with several molecules in its secretion including lysozyme (Lys). Lys has besides its antibacterial effects also antitumor and immunomodulation effects (7). In animal studies, testosterone replacement is reported to cause BPH (8-10).

## OBJECTIVE

The aim of the present study is to compare the effects and relevant histopathological changes of intra-prostatic BoNT-A and Lys gene injection into BPH tissue in a prospective controlled testosterone induced BPH rat model.

## MATERIALS AND METHODS

**Figure 1:**
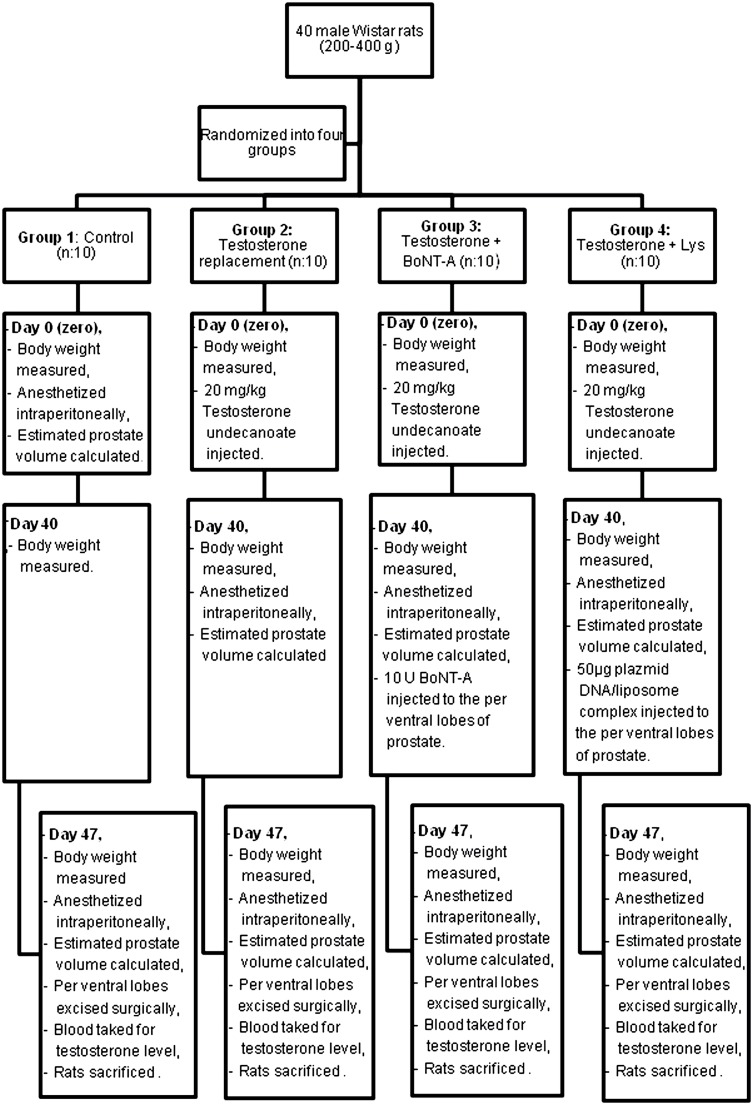
Experimental protocol.

Upon the approval of Suleyman Demirel University Animal Experiments Local Ethic Board, 40 male Wistar rats of 200-400g were included in the present study (Figure-1). In each cage 5 rats were kept in a room temperature of 22.4o Celsius. These were randomized into four Groups as following: Group 1: Control (n: 10), Group 2: Testosterone replacement (n: 10), Group-3: Testosterone+BoNT–A (n: 10), Group 4: Testosterone+Lys (n: 10).

At the beginning of the experiment, body weight of all rats was measured using a Pioneer made precision scale. This day was labeled as day zero. In order to anesthetize the rats in Group-1, 10mg/kg xylazin hydrochloride (Rompun^®^; Bayer Healthcare, Leverkusen, Germany) and 90mg/kg ketamine hydrochloride (Ketalar^®^; Pfizer, Istanbul, Turkey) were administered intraperitoneally. In supine position, right and left ventral lobes of the prostate were accessed via a midline abdominal incision under anesthesia. A Hart made mechanic compass was used for the three dimensions of the prostate. Estimated prostate volume (EPV) was calculated using ellipsoid formula “width x length x height x 0.5236”. Into the right gluteal muscle of Groups 2, 3, and 4 rats, 20mg/kg Testosterone undecanoate (Nebido^®^; Bayer Schering Pharma) was injected. A waiting period of 40 days was considered for BPH development.

40 days subsequent to testosterone injection, the body weight of all the rats was measured again. The dimensions of rat prostate lobes in Groups 2, 3, and 4 were measured via a midline abdominal incision access under anesthesia. 10U BoNT-A (BOTOX^®^; Abdi Ibrahim, Istanbul, Turkey) with 0.2mL isotonic saline was injected under anesthesia to the per ventral lobes of the rats in Group 3. 50μg plazmid DNA/liposome complex in 10μL solution was injected to the per ventral lobes of the rats in Group-4 under anesthesia.

Forty seven days following testosterone injection, the body weight of all rats was remeasured. As previously described, the prostate volumes of the rats were recalculated under anesthesia. Per ventral lobes of all animals were excised surgically, weighted using a precision scale, and recorded. Following blood sampling to measure testosterone levels, all rats were sacrificed via exsanguination. All tissues obtained were embedded in 4% paraformaldehyde (Merck) and stained with hematoxylin-eosin before histological evaluation and scoring using a light microscope. In order to obtain an objective comparative evaluation of the main histopathological findings a chart score protocol was used, which was described by Scolnik et al. (9). This protocol took into account the acinar morphology, such as crowding, intraluminal villosities, loss of basal nuclear polarity, and hyperplastic nodules, which were scored according to their degree of severity and distribution pattern. Tissues were categorized as normal and BPH. All animal's samples were analyzed immunohistochemically using Rabbit Active Caspaz-3 (Santa Cruz, sc-44976) and rabbit ABC Staining Kit (Santa Cruz, sc-2018). The active caspase 3 activity was used for apoptosis indicator. Asinus photos were taken at 400x enlargement in randomly chosen areas and 100 epithelial cells were counted for each sample. It was determined how many of these epithelial cells were positive for active caspase-3 staining. Rat blood testosterone levels were evaluated using Bmassay rat testosterone Elisa Kit.

Lysozyme gene preparation: pHM6 mammalian expression vector is a plasmide shuttle vector at 5442bp length. It can reproduce in many E. *coli* strains. Vector and E. *coli* XL1-Blue MRF bacteria had been obtained from Rize University, Medical Faculty, Department of Medical Biology and Genetics.

As the DNA molecules of the present pHM6 plasmide vector were limited in number, they were first transferred to E. coli and transformed recombinant bacteria stocks were formed. Using endotoxin-free Plazmide Midiprep kit, plasmide DNA isolations were achieved. The ideal form for transfection was plasmide DNA in supercoil form. DO-TAP/DNA complex (for 1μg DNA 5-10μg DOTAP) was used for liposomal transfection in line with the suggestions of the manufacturer. Lipid vesicles and pHM6m Lys DNA complexes were hence formed.

Data obtained were analyzed using SPSS 15.0 package program (Statistical Package for the Social Sciences for Windows). Measures of central tendency and data distribution were evaluated. The normality and homogeneity of the data were evaluated using Kolmogorov Smirnov Test and One-Way ANOVA (homogeneity of variance). In the comparison of independent Groups, Mann Whitney U Test, Friedman variant analysis and in dependent Groups Wilcoxon T Test and Kruskal-Wallis variant analyses were employed.

## RESULTS

The mean body weight of all the rats at day zero was 305.3±43.7 and at the 47^th^ day 327.2±47gr. There was no statistically significant difference among the Groups in terms of same day body weight (p=0.149). However, statistically significant differences in terms of body weight were present on comparing day zero and 47^th^ day within the groups (p<0.05). The rats had gained weight during the experiment. Only one rat died throughout the study (Group 4 no: 6). The mean testosterone level of the rats in Group 1 was 0.46±0.11ng/mL, Group 2 0.74±0.43ng/mL, Group-3 0.76±0.32ng/mL, and Group-4 0.77±0.29ng/mL. Blood testosterone levels of the testosterone injected Groups were significantly higher than Group 1 (p <0.05).

The mean EPV values of Group 1 on day zero and 47^th^ day and of Groups 2, 3, and 4 on the 40^th^ and 47^th^ day are presented in Table-1. There was no difference in the EPV of the rats in Group 1 at the beginning and at the end of the study (Table-1). No statistically significant difference was present in the testosterone injected Groups in terms of prostate growth on the 40^th^ day (Table-2). A statistically significant growth was seen in the EPV of the testosterone injected Groups on the 40^th^ day compared with Group 1 at the 47^th^ day.

**Table 1:** EPV measures made at different times and analysis of intra-group data.

	Gruplar	0 day	40 day	47 day	p
Group 1	RVPL	93.5±39.7	-	94.4±39.5	0.674
	LVPL	80.4±49.9	-	90.8±34.8	0.237
Group 2	RVPL	-	162.3±60	162.8±59.5	0.651
	LVPL	-	154.2±51.9	154.5±52.2	0.697
Group 3	RVPL	-	148.6±34.2	101.4±33	0.012
	LVPL	-	123.3±22.3	93.2±18.4	0.012
Group 4	RVPL	-	163.3±57.8	97.1±42.2	0.008
	LVPL	-	147.9±41	95.3±27.7	0.008

± = Standard deviation; p = *Wilcoxon T Tes*

**RvpL** = Right ventral prostate lobes

**LvpL** = Left ventral prostate lobes

**Measurement** = mm^3^ (cubic millimeter)

**Table 2:** EPV Comparative analyses of Group 1 at the 47^th^ day and Groups 2, 3, and 4 at the 40^th^ day.

	RVPL	LVPL
	p	p
Group 1-2	0.007	0.002
Group 1-3	0.007	0.009
Group 1-4	0.018	0.009
Group 2-3	0.583	0.754
Group 2-4	0.656	0.863
Group 3-4	0.713	0.614

**P** = *Mann Whitney U Test*

**RVPL** = Right ventral prostate lobes

**LvpL** = Left ventral prostate lobes

EPV of Groups 2, 3, 4 on the 47^th^ day were compared. There was a significant decrease in the EPV of the Groups that underwent a medical treatment compared with Group 2 (Table-3). 40^th^ and 47^th^ day intragroup EPV comparison revealed a statistically significant decrease in both the BoNT-A and the Lys treatment group (p=0.012).

**Table 3:** EPV Comparative analyses of the groups at the 47^th^ day.

	RVPL	LVPL
	p	p
Group 2-3	0.026	0.002
Group 2-4	0.014	0.006
Group 3-4	0.735	0.923

**P** = *Mann Whitney U Test*

**RVPL** = Right ventral prostate lobes

**LVPL** = Left ventral prostate lobes

Mean weight of the actual prostates upon excision is presented in Table-4. The comparison of Group 1 and 2 per ventral prostate lobe weight revealed a statistically significant difference (p=0.028, p=0.006). Testosterone leads to an increase in the prostate weight of the rats.

**Table 4:** Actual prostate weight of the groups.

	Group 1		Group 2		Group 3		Group 4	
	Right	Left	Right	Left	Right	Left	Right	Left
1	263	500	372	330	280	223	236	159
2	245	210	312	310	182	130	149	88
3	208	238	590	504	276	255	357	197
4	197	216	469	326	235	212	214	213
5	135	126	432	371	334	316	275	213
6	394	207	344	385	490	156	*	*
7	292	293	264	296	185	201	192	203
8	361	313	370	320	252	174	240	203
9	501	•	340	333	220	210	355	363
10	156	227	393	373	276	223	218	224

* = Ex;

• = Single lobe

**Measurement** = g (gram)

A statistically significant difference was present in the comparison of the actual per ventral prostate lobes of Group 2 with Groups 3 and 4 (p<0.05, p<0.05). On comparing the actual per ventral prostate lobe weights of Groups 3 and 4, no statistically significant difference was seen (p>0.05, p>0.05). Both BoNT-A and Lys treatment led to statistically significant losses in actual prostate sizes.

**Figure 2:**
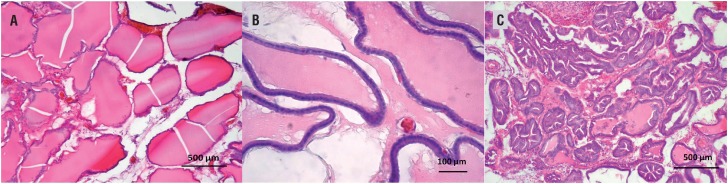
A) Normal prostate tissue. Regular acinus forms, homogeneous lumen secretion and connective tissue density. Hematoxylin-eosin (HE) stain, magnification 200X. scale Bar: 500μm.; B) The acini were surrounded with a thin stroma and the base membrane was thin and uninterrupted. Hematoxylin-eosin (He) stain, magnification 400X. scale Bar: 100μm.; c) Hyperplastic prostate tissue. columnar and overly curved acinus epithelium, irregular acinus and fibrotic connective tissue. Hematoxylin-eosin (He) stain, magnification 200X. scale Bar: 500μm.

The histological examination of Group 1 revealed that the acini preserved their lumen, structure, and space layout between acini. The acinar lumen was filled with homogenous acidophilic material. In some cases, very slight villous projections were observed. The glandular epithelium consisted of a single layer and was composed of small, round core, largely cubic or low columnar cells. The acini were surrounded with a thin stroma and the base membrane was thin and uninterrupted (Figures 2A and B). In Group 2, there were histological changes in line with BPH (Figure-2C). The epithelium was either cubic or cylindrical. The nucleus was either round or ovoid. There was mitotic activity in some of the cells. In some cases, there were either isolated or at multiple sites cell groupings in piling up formations. The basal membrane was thin and continuing. However, the H&E stained sections of the Groups 3 and 4 revealed some alteration at histological changes that was seen in Group 2.

Mean apoptotic number of cells in Group 1 was 26.1±7.3, in Group 2 22.9±5.2, in Group 3 34.3±5.6 and in Group 4 33.3±5.2. The number of apoptotic cells increased significantly comparing Groups 1 & 2 with the groups receiving treatment (p <0.005).

## DISCUSSION

The morphology and pathology of the dog prostate resembles human prostate most. However, dogs are in the category of higher vertebrates and limited in number in experimental animal laboratories and thus present increased costs. Therefore, in prostate studies rats and mice are preferred due to morphologic similarities despite the lack of an obstructive pattern. In rodent animals, the prostate lobes are classified as dorsal, ventral, lateral, and anterior (coagulating glands) lobes. Generally, in BPH studies the ventral lobe and in prostate cancer studies the dorsal and lateral lobe are preferred. The ventral lobe is a common site for prostatic hypertrophy and normal epithelial cells in the ventral lobe from aged rats are low cuboidal or flattened as compared to those in the dorsolateral lobe; recognition of a proliferative lesion by light microscopy is easy under low magnification (11). In the present we preferred the ventral lobes.

To form BPH model in rodent prostate, generally testosterone propionate, testosterone enanthate, estradiol, phenylephrine, growth factors, fetal urogenital sinus implants etc. are used (8-10, 12-15). In forming BPH model, the rat strain is also important. Scolnik et al. studied benign and atypical hyperplasia in the ventral prostate lobes of Wistar, Sprague-Dawley, Fischer and ACI/Ztm adolescent male rat strains in 1994 (9). They reported that Wistar is the most appropriate strain for the induction of prostate hyperplasia. In the present study, we preferred Wistar rats. In order to induce BPH 20mg/kg Testosterone undecanoate was used.

Comparison of rat prostate weight is among the different methods suggested in the literature to demonstrate BPH induction. In this method, the prostate weights are compared with the control Group or the Groups that were sacrificed at different times (8, 16-18). In the present study, we measured EPV (calculated using length, width, and height of per ventral prostate lobes) and APV (measured following surgical excision). EPV statistical analyses revealed no statistically significant difference in the initial and final values of control Group. We determine that the EPV of the testosterone injected Groups were higher than the control Group. The actual prostate weight of the testosterone injected Groups was higher than the control Group enabling us to attribute the increase to testosterone injection. Using 20mg/kg testosterone undecanoate increased the prostate weight of the rats 39%.

Another method used to demonstrate BPH induction, suggested in the literature, is histological evaluation (9-11, 19). The findings of the present study were in line with the findings in the literature.

Botulinum neurotoxin, discovered first in 1897, has been used as a therapeutic agent since 1977. BPH impact has been studied since 2003. BoNT-A injection to the urethra or bladder provides long term relief, 6 months or more, in lower urinary system dysfunctions (20). It was reported to inhibit urethral norepinephrine release and to cause atrophy in the prostate glands through selective denervation (20, 21). Human studies reported a decrease up to 40% in prostate size and a recurrence to the initial treatment size within approximately 18 months (2, 22, 23). 10 units of BoNT-A were used in the present study. BoNT-A treatment led to a EPV and actual prostate weights decrease up to 32.5% in rats leading to prostate apoptosis.

Lys is an important antimicrobial enzyme of the defense system. Besides its antimicrobial activities, it is also known to inactivate certain virus Groups, preserve cell membrane of mammalians, to increase polymorphonuclear leukocytes, macrophages and monocytes phagocytic/cytotoxic activity, to stimulate monocytes for analgesic, anti-tumoral, anti-metastatic, and anti-inflammatory activity, immunoglobulin production, and the induction of fosfolipid vesicular unity and thus increasing tumor cell immunogenicity (7, 24). The prostate is a secretory organ with several molecules in its secretion including Lys. Recent research has shown that lysozyme was a immunohistochemical marker in prostatic ductal adenocarcinomas (25, 26). In the present study, in order to evaluate the impact of Lys to BPH model intra-prostatic ~50μg plazmid DNA/liposome complex was applied. At the end of the study, Lys treatment led to a decrease in both EPV and actual prostate weight. The decrease in the prostate weight was determined as 38.7%.

Androgen effects on the prostate are mediated by dihydrotestosterone, which is converted from testosterone by the enzyme 5α-reductase. Two 5α-reductase inhibitors are available for clinical use: dutasteride and finasteride. 5α-reductase inhibitors act by inducing apoptosis of prostate epithelial cells leading to prostate size reduction of about 18-28% and a decrease in circulating PSA levels of about % 50 after 6-12 months of treatment (27).

Pharmacological therapies with 5α-reductase inhibitors have gained widespread acceptance as safe and effective treatments for BPH. But they have adverse effects such as reduced libido, erectile dysfunction and less frequently, ejaculation disorders such as retrograde ejaculation, ejaculation failure or decreased semen volume (27-29). Their effect on the serum PSA concentration needs to be considered for prostate cancer screening. Also, due to the slow onset of action, they are suitable only for long-term treatment. At this point, BoNT-A and Lys may be an alternative treatment option because of their safety, effectiveness, and quick onset effect. However, further studies are needed especially on humans to determine their safety and effectiveness.

## CONCLUSIONS

The present study was planned to answer the need for more effective non-invasive BPH treatment in the constantly aging male population throughout the world. The findings of the present study have shown that both BoNT-A and Lys treatment decreases the prostate weight in BPH. In terms of efficacy, there was no difference between both Groups.

Lys in BPH treatment has been first used in the present study. In rat BPH model, its prostate volume decreasing effect has been verified. As the present study proposes a novel treatment modality in BPH it should be verified in large randomized human experimental studies.

## ABBREVIATIONS

BPH: = Benign prostate hyperplasia
BoNT-A: = Botulinum toxin
EPV: = Estimated prostate volume
LUTS: = Lower urinary tract symptoms
Lys: = Lysozyme
